# Long-term outcomes of adult cryptogenic febrile infection–related epilepsy syndrome (FIRES)

**DOI:** 10.3389/fneur.2022.1081388

**Published:** 2023-01-04

**Authors:** Xiaojing Shi, Yuanyuan Wang, Xuan Wang, Xiaogang Kang, Fang Yang, Fang Yuan, Wen Jiang

**Affiliations:** ^1^Department of Neurology, Xijing Hospital, Fourth Military Medical University, Xi'an, China; ^2^Department of Neurology, The Second Affiliated Hospital of Guangzhou University of Chinese Medicine, Guangzhou, China

**Keywords:** febrile infection-related epilepsy syndrome, adult, new-onset refractory status epilepticus, refractory status epilepticus, long-term outcome, case series

## Abstract

**Background:**

Cryptogenic febrile infection-related epilepsy syndrome (FIRES) is a rare but catastrophic encephalopathic condition. We aimed to investigate the long-term outcome in adult cryptogenic FIRES.

**Methods:**

This was a retrospective study based on the prospective database in the neuro-intensive care unit of a tertiary hospital in China. Consecutive adult patients with cryptogenic FIRES between July 2007 to December 2021 were included. Long-term outcomes included function independence, the development of drug-resistant epilepsy (DRE), remote recurrent status epilepticus (SE), anti-seizure medications (ASMs), and changes in the brain Magnetic Resonance Imaging (MRI).

**Results:**

A total of 11 adult patients with cryptogenic FIRES were identified from 270 patients with SE. Four (36%) patients died in the hospital, with three of them withdrawing treatments, and one patient died 12 months after discharge. After the follow-up ranging from 12 to 112 months, 6 (55%) patients were still alive, and all of them achieved functional independence [modified Rankin Scale (mRS) 0-3]. 45% (5/11) patients developed DRE, 18% (2/11) had remote recurrent SE, and 55% (6/11) were on polytherapy with ASMs at the last follow-up. Most of the patients with initial normal or abnormal MRI had abnormalities in the hippocampus at follow-up, and most of the other MRI abnormalities found in the acute stage disappeared over time.

**Conclusion:**

The outcome of adult cryptogenic FIRES is daunting. More than one-third of patients die in the hospital. Survivors of cryptogenic FIRES may regain functional independence, but they usually develop DRE and receive polytherapy of ASMs for a long time.

## 1. Introduction

Febrile infection-related epilepsy syndrome (FIRES) is a rare but devastating encephalopathic condition. FIRES was first reported in children as “acute encephalitis with refractory, repetitive partial seizures” (AERRPS), and the term FIRES was first used by van Baalen et al. to report 22 children with prolonged or recurrent seizures after fever (1, 2). In recent years, FIRES has also been reported in adults, and this term has been used to emphasize the acute *de novo* presentation of refractory status epilepticus (RSE) without clearly identifiable acute or active causes (3–5). The clinical characteristics of FIRES are similar to those of new-onset refractory status epilepticus (NORSE), and both are thought to involve fulminant neurogenic inflammation in the brain. Based on the latest consensus, FIRES is considered a subcategory of NORSE that requires a prior febrile infection starting between 2 weeks and 24 h before the onset of RSE (6).

The exact pathophysiology of cryptogenic FIRES remains poorly understood. Some preliminary studies suggest that FIRES may involve a dysregulated innate immune system activation (6–8). The inflammatory cascade triggered by non-specific infections lowers the seizure threshold and precipitates seizures which in turn induce a massive neurogenic inflammatory response (9). Fulminant neurogenic inflammation and seizures become a vicious cycle that together contributes to recurrent seizures and status epilepticus. Besides the anti-seizure medications (ASMs), some immunomodulatory and anti-inflammatory therapies are used in patients with FIRES. High-dose steroids, intravenous immunoglobulin (IVIG), plasmapheresis, therapeutic hypothermia, and interleukin-1 receptor antagonist were reported to be partially efficacious in FIRES (9–13), but robust evidence is lacking.

In the acute phase, approximately 12% to 22% of patients cannot survive FIRES/NORSE (9, 14). In the long term, only 18% of children with FIRES regain normal cognitive function, and more than 90% develop refractory epilepsy requiring lifelong treatment (9, 15, 16). However, the long-term outcomes of adult patients with cryptogenic FIRES remain unknown. Due to the lack of related studies, we conducted a case series study to investigate the long-term outcome in adult cryptogenic FIRES, including functional independence, seizure outcomes, and changes in brain images.

## 2. Materials and methods

### 2.1. Study design

This study was a retrospective study based on a prospective database in the neuro-intensive care unit (NICU) at Xijing Hospital, China. This study was approved by the Ethics Committee of Xijing Hospital (KY20222115-C-1) and was conducted in compliance with Chinese laws and the Helsinki Declaration. Patients' consent was waived by the ethics committee.

### 2.2. Participants and definitions

The NICU database was searched between July 2007 to December 2021 for patients with cryptogenic FIRES. The inclusion criteria were as follows: (1) age 16 years or older; (2) status epilepticus (SE) refractory to at least 2 appropriately selected and dosed parenteral anti-seizure medications including a benzodiazepine (3, 17) with fever prior to the onset of RSE between 24 h and 2 weeks (6). Exclusion criteria were (1) active epilepsy or other preexisting relevant neurological disorder, and (2) SE due to a clear acute or active cause (6). Convulsive SE was defined as continuous or repetitive motor seizures without complete interictal recovery to clinical baseline (18). Non-convulsive SE (NCSE) was defined as a type of SE without prominent motor movements and manifested as neurological deficit, disturbance of consciousness and behavioral changes, and was diagnosed according to the Salzburg Consensus Criteria for NCSE (19).

### 2.3. Data collection

Collected clinical data included age, gender, medical history, prodromes, SE characteristics (worst type, duration, medications, treatment responses), findings of ancillary tests [cerebrospinal fluid (CSF) routine tests, CSF Metagenomic Next-Generation Sequencing (mNGS) test, CSF and peripheral blood autoantibody tests, peripheral blood Whole-Exome Sequencing test, tumor screening examinations, brain Magnetic Resonance Imaging (MRI), continuous electroencephalogram (EEG)], and treatments and duration in NICU. A prodrome was considered as any symptom prior to SE onset between 24 h and 2 weeks. SE semiology was classified according to the latest guidelines of SE (18). The following antibodies were tested in all patients: anti-NMDA-R, anti-CASPR2, anti-AMPA1-R, anti-AMPA2-R, anti-LGI1, anti-LGI2, anti-GABA2-R, anti-Hu, anti-Yo, anti-Ri, anti-Mn2, anti-CV2, anti-Amphiphysin, anti-ANNA-3, anti-Tr, anti-PCA-2, and anti-GAD.

### 2.4. SE monitoring and management

All the patients received continuous EEG monitoring (Solar 2000 N, Solar Electronic Technologies Co., Ltd., Beijing, China) to guide anti-seizure treatments and detect non-convulsive seizures. The management of SE was controlled by the same group of neurologists according to the clinical guidelines (20, 21): the first-line treatments were benzodiazepines; the second-line agents were intravenous sodium valproate, phenobarbital sodium and levetiracetam; and the third-line treatments were continuous infusions of anesthetics (midazolam or/and propofol).

### 2.5. Outcomes

Long-term outcomes included function independence, the development of drug-resistant epilepsy (DRE), remote recurrent SE, ASMs, and changes in the brain MRI. Functional independence was defined as a modified Rankin Scale (mRS) score of 0–3. DRE was defined as the failure of adequate trials of 2 tolerated and appropriately chosen and used ASMs (whether as monotherapies or in combination) to achieve sustained seizure freedom (22). Remote recurrent SE was defined as any episode of SE after hospital discharge (23). Outcomes were assessed by a trained neurologist based on clinical data obtained from routine consultations in the outpatient clinic and telephone interviews. The last follow-up ended in November 2022.

## 3. Result

**Figure 1:**
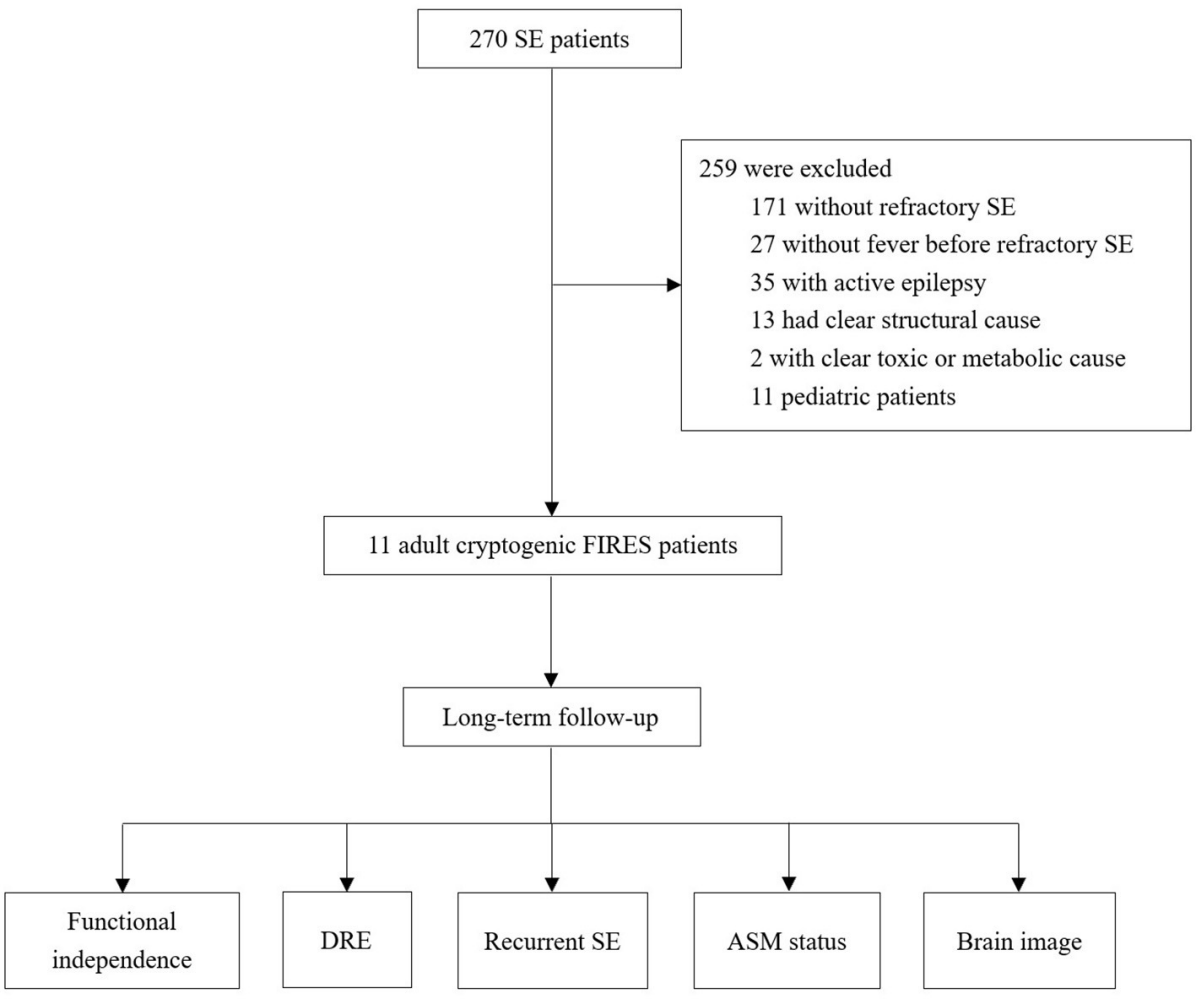
Flowchart of the study. SE, status epilepticus; FIRES, febrile infection-related epilepsy syndrome; DRE, drug-resistant epilepsy; AEM, anti-seizure medication.

We identified 11 cases fulfilling our criteria among 270 cases with SE (Figure 1). The clinical characteristics were summarized in Table 1 and presented in detail in Supplementary Tables 1, 2. Age ranged from 17 to 35 years, and the median age was 24 years. A female predominance was observed (74% vs. 36%). All the cases remained cryptogenic despite an extensive workup. The autoimmune antibody detection and CSF mNGS tests were negative in all patients. Most patients had negative findings in the Whole-exome sequencing test, and two patients had the heterozygous mutation in NFKB1 and ALDH7A1, respectively (Supplementary Table 2).

**Table 1:** Clinical characteristics of adult cryptogenic FIRES patients.

**Characteristics**	**All cases (*n* = 11)**
Age, years, median (IQR)	24 (21-30)
Male, *n* (%)	4 (36.4)
Time from fever to RSE, days, median (IQR)	5 (3-5)
Time form first seizure to SE, hours, median (IQR)	2 (1-5)
History of seizure, *n* (%)	1 (9.1)
**Prodrome, *n* (%)**	
Fever	11 (100.0)
Headache	5 (45.5)
Gastrointestinal symptoms	2 (18.2)
Behavioral changes	1 (9.1)
Confusion	1 (9.1)
**Worst SE type**, ***n*** **(%)**	
NCSE with coma	11 (100.0)
SE duration, days, median (IQR)	31 (15-77)
**NICU management**	
Number of anti-seizure medications, median (IQR)	6 (4-6)
Use of CIVADs, *n* (%)	10 (90.9)
Ketogenic diet, *n* (%)	3 (27.3)
Immunotherapies, *n* (%)	10 (90.9)
IV steroids, *n* (%)	9 (81.8)
IVIG, *n* (%)	8 (72.7)
Plasma exchange, *n* (%)	2 (18.2)
Mycophenolate mofetil, *n* (%)	1 (9.1)
Hypothermia, *n* (%)	2 (18.2)
Use of MV, *n* (%)	11 (100.0)
MV duration, days, median (IQR)	36 (10-77)
**EEG features of seizures, *n* (%)**	
Generalized onset	5 (45.5)
Lateralized onset, unilateral	3 (27.3)
Multifocal onset	3 (27.3)
Abnormal MRI, any, *n* (%)	3 (27.3)
NICU stay, days, median (IQR)	48 (22-78)
In-hospital death, *n* (%)	4 (36.4)

SE, status epilepticus; NCSE, non-convulsive status epilepticus; NICU, neurological intensive care unit; CIVADs, continous infusion of intravenous anesthetic drugs; MV, mechanical ventilation; IV, intravenous; IVIG, intravenous immunoglobulin.

The median time from fever to RSE was 5 days, and other prodromes included headache (46%), gastrointestinal symptoms (18%), behavioral changes (9%), and confusion (9%) (Table 1). All the patients had NCSE with coma. Generalized (46%) seizure onset was more common than lateralized (27%) and multifocal (27%). The median time from the first seizure to SE was 2 h, and one patient started with SE. All the cases were super refractory SE, and the median duration of SE was 31 days.

Patients received a median of 6 ASMs in NICU. Continuous infusion of anesthetics was used in 91% of patients, and mechanical ventilation was used in all the patients. Immunotherapies were used in 91% of patients, including intravenous steroids (82%), IVIG (73%), plasma exchange (18%), and mycophenolate mofetil (MMF) (9%). Ketogenic diet and hypothermia were used in 27% and 18% of patients, respectively. The median length of NICU stay was 48 days. Four (36%) patients died in the hospital, and treatments were withdrawn in three of them at the request of their families: Patient 1 developed sepsis and severe liver and heart failure, Patient 3 developed severe anemia (hemoglobin 28 g/L) despite receiving blood transfusions for 13 consecutive days (possibly due to visceral hemorrhage), and Patient 4 developed refractory septic shock.

**Figure 2:**
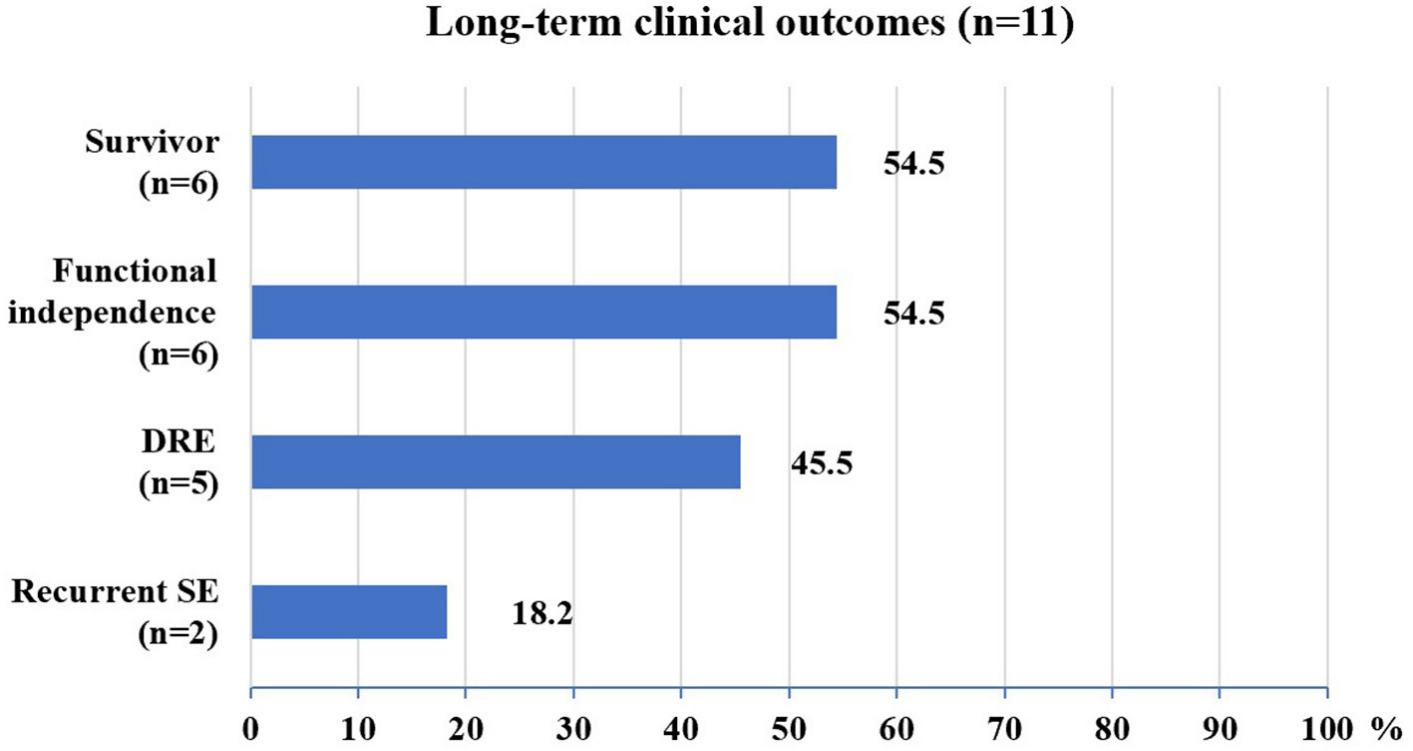
Long-term clinical outcomes of FIRES patients. DRE, drug-resistant epilepsy; SE, status epilepticus.

**Figure 3:**
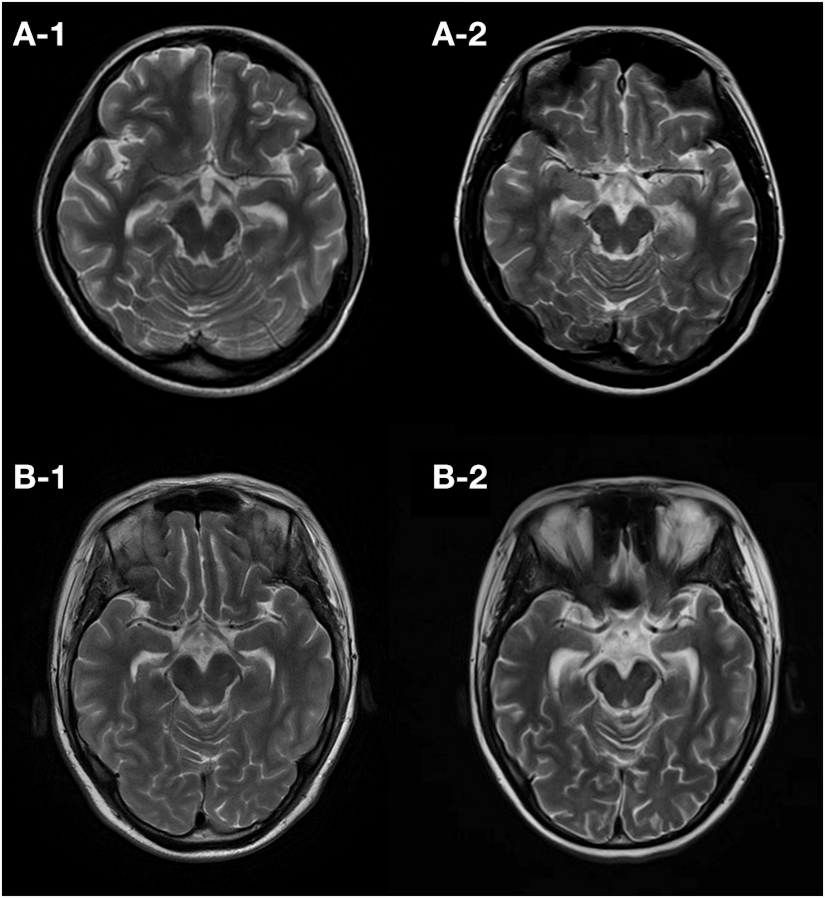
Follow-up T2 images of patients developing atrophy. Patient 7 had an initial normal brain MRI (10 days from onset) and showed hippocampal atrophy 50 days **(A-1)** and 72 months **(A-2)** later. Patient 11 also had an initial normal MRI (2 days from onset) and showed hippocampal atrophy 3 months **(B-1)** and 16 months **(B-2)** later.

The median follow-up in this study was of 20 months, ranging from 12 to 112 months. At the last follow-up, 5 (45%) patients died (4 died in the NICU, and 1 died 12 months after NICU discharge), and 6 (55%) patients were alive (Figure 2). All of these FIRES survivors achieved functional independence. Five patients (45%) had recurrent seizures (mostly generalized) after NICU discharge with a frequency of 3–180 seizures per month (Supplementary Table 3). All of these patients developed DRE (45%), and 2 (18%) had remote recurrent SE (Figure 2). Among 7 NICU survivors, 6 (86%) patients were on polytherapy with ASMs at the last follow-up. Levetiracetam (86%) and phenobarbital (71%) were more commonly used than other ASMs, such as valproate, topiramate, and carbamazepine (Table 2). Eight (73%) patients had normal brain MRI during the acute phase, of whom 4 died in NICU and the other 4 developed brain MRI abnormalities (mainly in the hippocampus) at follow-up (Table 3). Three (27%) patients had abnormal MRIs during the acute phase. MRI lesions disappeared completely in 1 patient after 6 months and recovered partially in 2 patients after 1–5 months. Of the 7 patients with follow-up MRIs, 4 had abnormalities in the hippocampus, 1 had hydrocephalus, 1 had abnormal signals in temporal and occipital lobes, and 1 had a normal brain MRI. Among the four patients with abnormalities in the hippocampus, two patients had T2/FLAIR hyperintensity in the hippocampus, and two patients had hippocampal atrophy (one of them had global brain atrophy). The follow-up MRI images of these two patients were presented in Figure 3.

**Table 2:** ASM status of NICU survivors at the end of follow-up.

	**NICU survivors (*n* = 7)**
**Treatment status**, ***n*** **(%)**	
Monotherapy	1 (14.3)
Polytherapy	6 (85.7)
**ASM, *n* (%)**	
LEV	6 (85.7)
PB	5 (71.4)
TPM	3 (42.9)
CZP	3 (42.9)
VPA	2 (28.6)
LTG	2 (28.6)
PER	2 (28.6)
CBZ	1 (14.3)
OXC	1 (14.3)
LCM	1 (14.3)

ASM, anti-seizure medication; LEV, levetiracetam; PB, phenobarbital; TPM, topiramate; CZP, clonazepam; VPA, valproate; LTG, lamotrigine; PER, perampanel; CBZ, carbamazepine; OXC, oxcarbazepine; LCM, lacosamide.

**Table 3:** The initial and follow-up brain MRIs.

**Patient**	**Initial**		**Follow-up**	
	**Time from onset, days**	**Location of abnormalities**	**Time from onset, months**	**Location of abnormalities**
1	1	Normal	Death	-
2	10	Bilateral cingulate gyrus, frontotemporal and insular cortex	6	Normal
3	1	Normal	Death	-
4	2	Normal	Death	-
5	1	Corpus callosum, bilateral frontal parietal islands occipital lobe	11	Hydrocephalus
6	1	Normal	8	Bilateral hippocampi
7	10	Normal	72	Hippocampal atrophy and global brain atrophy
8	1	Normal	Death	-
9	1	Corpus callosum, bilateral hippocampi	1	Bilateral hippocampi
10	1	Normal	1	Bilateral temporal lobes and insulas, left occipital lobe
11	2	Normal	16	Bilateral hippocampal atrophy

## 4. Discussion

This is the first study reporting the long-term outcome of adult patients with cryptogenic FIRES. This case series study showed a long-term mortality rate of 45% for cryptogenic FIRES, with most survivors achieving functional independence but developing DRE, receiving polytherapy of ASMs, and developing hippocampal abnormalities in the long term.

The in-hospital mortality of adult FIRES in this study was 36%, much higher than that reported in pediatric studies (24). A multicenter study of 77 children with FIRES reported in-hospital mortality of 12% (9), and another study including 16 FIRES children from the pSERG (the United States Pediatric Status Epilepticus Research Group) database reported in-hospital mortality of 19% (25). The pSERG cohort also found that FIRES had a more prolonged course and worse outcomes compared to other types of NORSE in children, which is consistent with our findings in adults. In a multicenter study of 125 adults with NORSE, in-hospital mortality was lower for all-type NORSE (22%), NORSE with a clear cause (18%), and cryptogenic NORSE (27%) than for cryptogenic FIRES in this study (14). Their study also reported shorter durations of SE and ICU stay for all-type NORSE, NORSE with a clear cause, and cryptogenic NORSE compared to cryptogenic FIRES (36%) in this study. In a study of 26 NORSE adults (73% were cryptogenic), in-hospital mortality (23% vs. 36%), SE duration (17 vs. 31 days), and length of ICU stay (32 vs. 48 days) were also lower than in this study (26). These studies indicate that cryptogenic NORSE/FIRES is more severe than NORSE with a clear cause, and cryptogenic FIRES is even more severe than cryptogenic NORSE.

The long-term outcomes of adult FIRES were previously reported in a German study of 6 cases (27). In their study, only 1 case was cryptogenic, and the rest were due to autoimmune or parainfectious encephalitis. All these 6 patients achieved functional independence (mRS ≤ 3) but were found to have refractory epilepsy, brain atrophy, and severe memory impairment. Although the severity (e.g., SE duration and worst type) of FIRES patients in their study is unknown, their findings regarding functional outcomes and the development of refractory epilepsy are consistent with ours. With or without a clear cause, most adult FIRES survivors can regain the ability to move unassisted. However, they usually develop MRI abnormalities in the hippocampus or mesial temporal lobes, and their quality of life is severely affected due to refractory epilepsy.

Patients with NORSE are more likely to develop DRE than patients with SE of all causes. In this study, 5 of 7 (71%) NICU survivors developed DRE, and 2 of them experienced remote recurrent SE. Two cohort studies of adult NORSE patients also showed high DRE rates of 75–80% in survivors (28, 29), while only 37% of patients with SE of all causes developed DRE (30). Children with FIRES have an even higher DRE rate of 93% (9). One possible reason is that there are more survivors of FIRES in children than in adults, and these survivors usually develop DRE in the long term. However, there is not much difference in the risk of remote recurrent SE between FIRES (29%) and all-cause SE (32%) (31).

Approximately 73% of patients with cryptogenic FIRES in this study had normal brain MRI scans in the acute phase, which is consistent with a rate of 61% in pediatric patients with FIRES (32). Lesions of FIRES on MRI usually involve the temporal lobe, basal ganglia, insula, and thalamus (27, 32). Previous case studies also found that T2/FLAIR hyperintense lesions appeared in bilateral claustrum on average 10 days after SE (29, 33). However, no claustrum abnormality was observed in our patients. In this study, most of the patients with initial normal or abnormal MRI had abnormalities in the hippocampus at follow-up, and most of the other MRI abnormalities found in the acute stage disappeared over time. In addition, previous pediatric and adult cases of FIRES showed that generalized brain atrophy and mesial temporal sclerosis were also frequently found in the chronic phase (27, 32).

Whether and how to give immunomodulatory therapies after the acute phase of cryptogenic FIRES is a clinical dilemma. DRE and cognitive impairment are major challenges after NICU discharge for patients, their families, and clinicians. Initially, we focused on treating DRE with various ASMs and did not give immunomodulatory therapies after NICU discharge. Recently, we tried immunomodulatory treatments for patients with cryptogenic NORSE/FIRES after NICU discharge, on the assumption that they may involve dysregulated innate immune system activation. Some patients were given MMF after the use of high-dose methylprednisolone and IVIG in the acute phase and continued to receive MMF for 1–2 years after discharge from NICU. Some patients received sirolimus and/or repeated cycles of IVIG after NICU discharge. The cycles of IVIG and the duration of sirolimus depend on the seizure outcomes. However, patients were often reluctant to continue treatment if their seizure control did not improve after 1–2 cycles of IVIG or after 3 months of sirolimus. In this study, Patient 6 received MMF and Patient 11 received repeated cycles of IVIG and sirolimus after discharge from NICU, but neither of them had improved seizure control. In addition, we are also gaining experience with rituximab, interleukin-1 receptor antagonists, and interleukin-6 antagonists in the post-acute phase of FIRES. The use of immunomodulatory therapy for cryptogenic FIRES after NICU discharge remains disputable and requires further randomized studies.

The limitations of this study include the single-center design and small sample size which reduce its generalizability. However, FIRES is a very rare condition, and this calls for further multicenter and international studies. The timing of brain MR in this study was highly variable, and it is unclear whether the MRI abnormalities found at follow-up were caused by FIRES or DRE. In addition, neuropsychological outcomes may also affect the quality of life, such as intellectual impairments and mental state, but they were not assessed in this study.

## 5. Conclusion

The outcomes of adult patients with cryptogenic FIRES are daunting. More than one-third of patients die in the hospital. Survivors of cryptogenic FIRES may regain functional independence, but they usually develop DRE and receive polytherapy of ASMs for a long time. Future studies are needed to answer many open questions on this clinical challenge.

## Data availability statement

The original contributions presented in the study are included in the article/Supplementary material, further inquiries can be directed to the corresponding authors.

## Ethics statement

The studies involving human participants were reviewed and approved by the Ethics Committee of Xijing Hospital. Written informed consent from the participants' legal guardian/next of kin was not required to participate in this study in accordance with the national legislation and the institutional requirements.

## Author contributions

XS and YW: data collection, interpretation, and analysis. XK and FYa: data checking. XS and FYu: drafting manuscript. YW, XW, and FYu: drafting review. WJ and FYu: study design and critical revision. WJ: study supervision and obtaining funding. All authors approved the final version.
