# Supplementary material for: Long-term outcomes of adult cryptogenic febrile infection–related epilepsy syndrome (FIRES)
Source: Front Neurol. 2023 Jan 4;13:1081388. doi: 10.3389/fneur.2022.1081388 (PMC9848432; doi:10.3389/fneur.2022.1081388)
Supplement: Supplementary file 1 [file Table_1.docx]

Supplementary Table 1. Clinical profiles in the acute phase.

| Patient | Age/Gender | Days from fever to RSE | Hours from first seizure to SE | Prodromes | Seizure history | Worst seizure type | SE duration, days | NICU stay, days | MV duration, days | Continuous infusions | ASMs | Other therapy |
| --- | --- | --- | --- | --- | --- | --- | --- | --- | --- | --- | --- | --- |
| 1 | 35/M | 5 | 3 | Fever, headache | Normal | NCSE with coma | 10 | 10 | 10 | MDZ, PB, KT, propofol, vecuronium | DZP, LEV, TPM | Steroid, hypothermia |
| 2 | 20/F | 2 | 2 | Fever | Normal | NCSE with coma | 18 | 22 | 18 | PB | DZP, TPM, LEV, LTG, VPA, PB | - |
| 3 | 24/F | 3 | 1 | Fever, headache, behavioral changes | Normal | NCSE with coma | 77 | 78 | 77 | MDZ, PB, propofol | VPA, CBZ, LEV, TPM, CZP, DZP, PB | IVIG, steroid, KD |
| 4 | 28/M | 3 | 4 | Fever | Normal | NCSE with coma | 58 | 59 | 58 | MDZ, KT, PB, propofol | TPM, LEV, CBZ, VPA, DZP, PB | IVIG, KD, plasma exchange |
| 5 | 21/F | - | 1 | Fever | Febrile convulsions | NCSE with coma | 208 | 226 | 210 | MDZ, PB, propofol | CZP, LEV, LCM, GBP, TPM, PER, VPA, DZP, PB | IVIG, steroid, plasma exchange, hypothermia |
| 6 | 23/M | 5 | 72 | Fever, vomiting | Normal | NCSE with coma | 21 | 48 | 36 | MDZ | VPA, LEV, CBZ, CZP | IVIG, steroid, mycophenolate mofetil |
| 7 | 17/F | 5 | 0.5 | Fever | Normal | NCSE with coma | 31 | 48 | 30 | MDZ, PB | LEV, CZP, CBZ, VPA, DZP, PB | IVIG, steroid |
| 8 | 22/F | 5 | 1 | Fever, headache | Normal | NCSE with coma | 78 | 78 | 78 | MDZ, PB, propofol | TPM, LEV, CBZ, CZP, DZP, PB | IVIG, steroid, KD |
| 9 | 27/F | 4 | 5 | Fever, headache | Normal | NCSE with coma | 15 | 18 | 3 | MDZ | LEV, TPM, VPA, CBZ, PB | IVIG, steroid |
| 10 | 30/M | 5 | 48 | Fever, headache | Normal | NCSE with coma | 11 | 27 | 12 | MDZ, PB | CBZ, VPA, TPM, LEV | Steroid |
| 11 | 30/F | 5 | 0 | Fever, vomiting, confusion | Normal | NCSE with coma | 45 | 78 | 45 | MDZ, PB, propofol | VPA, OXC, LEV, CZP, LTG, PB | IVIG, steroid |

Abbreviations: ASMs = antiseizure medications; CBZ = carbamazepine; CH = chloral hydrate; CSF = cerebrospinal fluid; CZP = clonazepam; DZP = diazepam; GBP = gabapentin; IVIG = intravenous immunoglobulin; KD = ketogenic diet; KT = ketamine; LCM = lacosamide; LEV = levetiracetam; LTG = lamotrigine; MDZ = midazolam; MV = mechanical ventilation; NCSE = nonconvulsive status epilepticus; OXC = oxcarbazepine; PB = phenobarbital; PER = perampanel; RSE = refractory status epilepticus; SE = status epilepticus; TPM = topiramate; VPA = valproate.
