# Supplementary material for: Long-term outcomes of adult cryptogenic febrile infection–related epilepsy syndrome (FIRES)
Source: Front Neurol. 2023 Jan 4;13:1081388. doi: 10.3389/fneur.2022.1081388 (PMC9848432; doi:10.3389/fneur.2022.1081388)
Supplement: Supplementary file 2 [file Table_2.docx]

Supplementary Table 2. Ancillary examinations.

| Patient | CSF WBC count, cells/μL | CSF Protein levels, mg/dL | CSF mNGS | Autoantibody evaluations | Gene examination | Tumor screening | Seizures in continuous EEG |
| --- | --- | --- | --- | --- | --- | --- | --- |
| 1 | 0 | 24.1 | Negative | Negative | Normal | Negative | Generalized onset |
| 2 | 18 | 60.0 | Negative | Negative | Normal | Negative | Generalized onset |
| 3 | 200 | 54.3 | Negative | Negative | Normal | Negative | Lateralized onset, unilateral |
| 4 | 3 | 40.0 | Negative | Negative | Normal | Negative | Generalized onset |
| 5 | 60 | 55.0 | Negative | Negative | Heterozygous mutation in NFKB1 | Negative | Lateralized onset, unilateral |
| 6 | 2 | 35.0 | Negative | Negative | Normal | Negative | Multifocal onset |
| 7 | 2 | 10.0 | Negative | Negative | Normal | Negative | Lateralized onset, unilateral |
| 8 | 10 | 53.8 | Negative | Negative | Normal | Teratoma | Generalized onset |
| 9 | 5 | 40.0 | Negative | Negative | Normal | Negative | Generalized onset |
| 10 | 10 | 40.0 | Negative | Negative | Normal | Negative | Multifocal onset |
| 11 | 2 | 67.0 | Negative | Negative | Heterozygous mutation in ALDH7A1 | Negative | Multifocal onset |

mNGS = Metagenomic Next-Generation Sequencing.
