# Supplementary material for: Long-term outcomes of adult cryptogenic febrile infection–related epilepsy syndrome (FIRES)
Source: Front Neurol. 2023 Jan 4;13:1081388. doi: 10.3389/fneur.2022.1081388 (PMC9848432; doi:10.3389/fneur.2022.1081388)
Supplement: Supplementary file 3 [file Table_3.docx]

Supplementary Table 3. Clinical outcomes.

| Patient | mRS  at discharge | mRS at last follow-up | Length of follow-up, month | Recurrent Seizures | Seizure frequency, per month | Recurrent SE | DRE | ASMs | Other therapy |
| --- | --- | --- | --- | --- | --- | --- | --- | --- | --- |
| 1 | 6 (withdrawal of treatment) | - | In-hospital death | NA | NA |  |  |  |  |
| 2 | 4 | 1 | 112 | Generalized & focal | 5-6 | No | Yes | PB, TPM, VPA, LEV | No |
| 3 | 6 (withdrawal of treatment) | - | In-hospital death | NA | NA |  |  |  |  |
| 4 | 6 (withdrawal of treatment) | - | In-hospital death | NA | NA |  |  |  |  |
| 5 | 4 | 6 | 20 | Generalized & focal | 120-180 | Yes | Yes | PB, PER, CZP, LCM | No |
| 6 | 1 | 1 | 13 | Focal | 4 | No | Yes | LEV, OXC, LTG, CZP | Mycophenolate mofetil |
| 7 | 3 | 2 | 75 | Generalized | 3 | Yes | Yes | PB, TPM, CBZ, LEV | taVNS |
| 8 | 6 | - | In-hospital death | NA | NA |  |  |  |  |
| 9 | 1 | 0 | 84 | Seizure-free | 0 | No | No | PB, LEV | No |
| 10 | 1 | 1 | 12 | Seizure-free | 0 | No | No | LEV | No |
| 11 | 3 | 2 | 13 | Generalized | 11 | No | Yes | LEV, VPA, TPM, PB, LTG, CZP, PER | IVIG, sirolimus |

Abbreviations: ASMs = antiseizure medications; CBZ = carbamazepine; CZP = clonazepam; DRE = drug-resistant epilepsy; IVIG = intravenous immunoglobulin; LCM = lacosamide; LEV = levetiracetam; LTG = lamotrigine; mRS = modified Rankin Scale; OXC = oxcarbazepine; PER = perampanel; PB = phenobarbital; SE = status epilepticus; taVNS = transcutaneous auricular vagal nerve stimulation; TPM = topiramate; VPA = valproate.
